# Epidemic of multiple *Treponema pallidum* strains in men who have sex with men in Japan: efficient multi-locus sequence typing scheme and indicator biomarkers

**DOI:** 10.1186/s12981-024-00663-y

**Published:** 2024-10-16

**Authors:** Wakana Sato, Ayako Sedohara, Michiko Koga, Yu Nakagama, Hiroshi Yotsuyanagi, Yasutoshi Kido, Eisuke Adachi

**Affiliations:** 1 Department of Laboratory Medicine, IMSUT Hospital of The Institute of Medical Science, The University of Tokyo, Tokyo, Japan; 2 Department of Infectious Diseases and Applied Immunology, IMSUT Hospital of The Institute of Medical Science, The University of Tokyo, 4-6-1 Shirokanedai, Minato-ku, Tokyo, 108-8639 Japan; 3 Division of Infectious Diseases, Advanced Clinical Research Center, The Institute of Medical Science, The University of Tokyo, Tokyo, Japan; 4 Department of Virology and Parasitology, Graduate School of Medicine, Osaka Metropolitan University, Osaka, Japan

**Keywords:** Multi-locus sequence typing, *Treponema pallidum subspecies pallidum*, Syphilis, *Treponema pallidum* subsp. *endemicum*, Bejel

## Abstract

**Background:**

The challenges in culturing Treponema pallidum have hindered molecular-biological analysis. This study aims to establish a molecular epidemiological analysis of syphilis among Japanese men who have sex with men (MSM) and to investigate the relationship between bacteremia and associated pathophysiology.

**Methods:**

We used whole blood specimens from syphilis-diagnosed individuals in Tokyo, collected between February 2019 and June 2022. All individuals were MSM, and most were people with HIV (97.2%). We used a multi-locus sequence typing (MLST) scheme for epidemiological analysis. Sequences for MLST (*TP0136*,* TP0548*, and *TP0705*) were obtained.

**Results:**

Out of 71 whole blood samples, 26 samples (36.6%) were positive for *TP0136*, and we sequenced three loci for MLST in 22 samples (31.0%). The most frequently detected sequence type (ST) was ST3 (*n* = 9), followed by ST6 (*n* = 6). Phylogenetic analysis revealed that 12 samples belonged to the SS14-like group (60%), and 8 samples belonged to the Nichols-like group (40%). *Treponema pallidum* subsp. *endemicum* (TEN), the cause of bejel was detected in three samples (12%). There was a significant association between *TP0136* detection rate and C- reactive protein (CRP) (77.0% at a cut-off:0.5 mg/dL).

**Conclusion:**

Both SS14-like and Nichols-like strains were circulating concurrently, and TEN could have been sexually transmitted among MSM with HIV. Elevated CRP may indicate the presence of the pathogen in the blood.

**Supplementary Information:**

The online version contains supplementary material available at 10.1186/s12981-024-00663-y.

## Introduction

*Treponema pallidum (T.pallidum)* subsp. *pallidum* (TPA), the causative agent of syphilis, has long been known to be prevalent among men who have sex with men (MSM). Currently, the number of new cases of syphilis is estimated to be 6 million worldwide [1]. In Japan, in particular, heterosexual transmission among young women has increased significantly in recent years [2]. TPA poses a challenge for clinical use due to the difficulty in culturing it. Edmondson et al. reported successful long-term culture of TPA using the rabbit epithelial cell co-cultivation system; however, this system has not been readily available in hospital laboratories worldwide [3]. Endemic treponemas (yaws, bejel, and pinta), classified as neglected tropical diseases (NTDs) and recognized as non-sexually transmitted syphilis, are caused by microbes *T. pallidum* subsp. *pertenue* (TPE), *T. pallidum* subsp. *endemicum* (TEN), and *T. pallidum* subsp. *carateum.* These are genetically highly similar to TPA and cannot be differentiated from it using the serological diagnostic methods. While it is possible that endemic treponemas are sexually transmitted and lead to epidemics, investigating this requires establishing a genetic diagnostic method capable of distinguishing them.

The diagnosis of syphilis involves both treponemal and nontreponemal tests [4]. The nontreponemal test measures antibodies against lipoidal material released by damaged host cells, such as cardiolipin; the nontreponemal test assesses the effect of treatment [5]. Although clinical diagnostic methods utilizing direct detection targets like *polA* and *tpp47* have been proposed [6, 7], these methods became less common due to the difficulty to culture TPA [3]. Moreover, molecular epidemiological analysis faced similar challenges. Samples for molecular epidemiological analysis are limited. Chancre specimens are suitable for molecular epidemiological analysis, but patients with primary syphilis who present with chancres represent only a small fraction of those with syphilis encountered in the typical outpatient setting for MSM. On the other hand, whole blood is less sensitive, and whole-genome sequence (WGS) analysis has been difficult [8]. There is a need for molecular epidemiological analysis methods that can be performed with easily collected blood samples, enabling phylogenetic analysis with sufficient discriminatory power. Multi-locus sequence typing (MLST) has been proposed for molecular epidemiological analysis of TPA. Grillová et al. developed a new MLST with high resolution power using three loci: *TP0136*,* TP0548*,* TP0705*, achieving enough discriminatory power to distinguish TPA from other treponemal subspecies and differentiate between the two TPA clades SS14 and Nichols [9]. Although whole genome sequencing has recently been an alternative option for molecular epidemiology, simpler MLST has more advantages in terms of cost, a subset of samples, and a growing public treponemal MLST database for storing and analyzing typing data.

Ultimately, challenges in molecular epidemiological studies, genetic diagnosis, and the establishment of markers other than antibody titers in syphilis, where the pathogen presents significant challenges to culture, arise from the difficulty in collecting specimens containing genomic information. To address this, we explored the feasibility of utilizing blood samples, which can be collected from all patients with syphilis, including those with asymptomatic early-stage syphilis. This study overcomes technical challenges by typing multiple genetic polymorphisms of *T. pallidum* using the MLST method and utilizing blood samples. Our aim is to identify the prevalent *T. pallidum* species in Japanese MSM with HIV, determine factors responsible for changes in the infected population, and prevent the spread of infection.

## Materials and methods

### Clinical samples

Clinical samples were collected from the individuals with syphilis diagnosed by clinical symptoms and serological tests in IMSUT hospital, the Institute of Medical Science, The University of Tokyo between February 2019 to June 2022. All cases were above 10 U/mL of TPLA and 1.0 RU of RPR. We reviewed routinely collected clinical records of the patients, including gender, age, HIV status, and the stage of syphilis, along with the results of serological tests. Ethics approval was granted by the ethics board of the Institute of Medical Science, University of Tokyo (2020-79-0324). Written consent was obtained from all participants.

### DNA extraction, purification, and nested PCR

DNA was extracted from whole blood samples of participants using QIAamp DNA Mini Kit (Qiagen, Germany) following the manufacture’s protocol. The elution (100 µl) was concentrated to a 20 µl solution using Ethachinmate (Nippon gene, Japan).

PCR primers as described previously by Grillová et al. [9], were employed. Optimal PCR conditions were verified using synthetic DNA (Azenta). Modified PCR procedure was used as below: For the first PCR, the total volume was 50 µl, with each reaction consisting of 15 µl of the concentrated template solution, 25 µl of KOD one PCR master mix (TOYOBO, Japan), and 1 µl of each primer (10 µM). The first PCR conditions were as follows: 95 ℃ for 2 min, followed by 5 cycles at 98 ℃ for 10 s, 66 ℃ for 10 s, 5 cycles at 98 ℃ for 10 s, 63 ℃ for 10 s, 5 cycles at 98 ℃ for 10 s, 60 ℃ for 10 s, 5 cycles at 98 ℃ for 10 s, 57 ℃ for 10 s, and finally, 68 ℃ for 1 min. For the second PCR, the total volume was 20 µl, with each reaction containing 5 µl of the first PCR product, 10 µl of KOD one PCR master mix (TOYOBO, Japan), and 0.4 µl of each primer. The second PCR conditions were: 95 ℃ for 2 min, followed by 5 cycles at 98 ℃ for 10 s, 63 ℃ for 10 s, 5 cycles at 98 ℃ for 10 s, 60 ℃ for 10 s, and finally, 68 ℃ for 5 min.

In case of *TP0705* and *TP0548* PCR negative despite *TP0136* PCR positive, direct pellet PCR was performed increase the sensitivity of PCR detection. The elution (50 µl) was concentrated using Ethachinmate, and the pellet was dissolved directly in PCR ingredients. The solutions were incubated for 10 min at room temperature. PCR products were purified using the QIAquick purification kit (Qiagen, Germany) following the manufacturer’s protocol.

### Multi-locus sequence typing and phylogenetic analysis

The purification products were used to determine the sequence by Sanger sequencing. MLST was performed for three loci, *TP0136*,* TP0548 and TP0705*. The sequences were uploaded to PubMLST BIGSdb of TPA for decision of allele number and Sequence Type (ST) [10]. The phylogenetic tree was constructed using MEGA X with the Maximum Likelihood method and bootstrap test [11]. The evolutionary history was determined employing the Maximum Likelihood method and the Tamura-Nei model [12]. The percentage of trees where the associated taxa clustered together is presented next to the branches. Initial trees for the heuristic search were automatically generated by applying Neighbor-Join and BioNJ algorithms to a matrix of pairwise distances estimated through the Maximum Composite Likelihood (MCL) approach. The topology with the highest log-likelihood value was then selected. This analysis comprised 9 nucleotide sequences, resulting in a total of 2577 positions in the final dataset. The concatenated sequences were downloaded from PubMLST BIGSdb.

### Investigation of biomarkers associated with the *TP0136* detection

We initially tested for *TP0136*, and the PCR-positive samples were subsequently tested for TP0548 and TP0705. Percentage of samples sequenced as *TP0136* (i.e., *TP0136* detection rate) was examined in relation to the following biomarkers: *T. pallidum* latex agglutination (TPLA), rapid plasma regain (RPR), and C-reactive protein (CRP). The tests were performed using RAPIDIA Auto TP (FUJIREBIO Inc., Japan), LASAY Auto RPR (SHIMA Laboratories Co., Ltd, Japan), and N-assay LA CRP-T Nittobo (Nittobo Medical Co., Ltd., Japan) on LABOSPECT 006 (Hitachi Hi-Tech Cp., Ltd., Japan), respectively. These tests were conducted on whole blood samples from which DNA was extracted, and serum was obtained on the same day. For TPLA, cutoffs of 100 U/mL, and 1000 U/mL; for RPR, cutoffs of 10 RU, and 100 RU; and for CRP, cutoffs of 0.3 mg/dL, 0.5 mg/dL, and 1.0 mg/dL were examined for correlation with *TP0136* detection rate.

### Statistical analysis

Demographic and clinical parameters are expressed as the median and interquartile range (IQR); qualitative variables are expressed as frequencies and percentages. Distributed variables were compared using the Mann–Whitney test; Differences in factors presented as percentages were tested using the Fisher’s exact test. The correlation between the quantitative values of RPR, TPLA, and CRP with the detection rate of *TP0136* was calculated using the Cochran-Armitage test for trend. We used Fisher’s exact test to evaluate the association between the biomarkers and the *TP0136* detection rate at each cutoff. Statistical significance was defined as two-sided *p* < 0.05. Statistical analyses were performed using Prism 9 (GraphPad Software).

## Results

### Characteristics of individuals with syphilis and clinical samples

We collected 71 samples, one from each untreated individual with syphilis. The demographic and clinical characteristics of the cases are summarized in Table 1. All individuals were male, and most of them were people with HIV (PWH) (97.2%). The median age of the participants was 41.

**Table 1:** Clinical characteristics of cases with syphilis

		All cases		Cases that were not accessible for MLST		Fully-typed cases by MLST		*P* value
Number of cases		71		51		20		
Median age, yr (IQR)		43	(37–49)	41	(34–48)	44	(37–50)	0.37^†^
HIV infection, n (%)		69	(97.2)	51	(100)	18	(90.0)	0.08^‡^
Stage of syphilis, n (%)	Primary syphilis	7	(9.9)	3	(7.8)	4	(20)	0.09^‡^
	Secondary syphilis	23	(32)	15	(29)	8	(40)	0.41^‡^
	Early Asymptomatic syphilis	35	(49)	28	(54)	7	(35)	0.19^‡^
	Neurosyphilis^*^	6	(8.5)	3	(5.9)	3	(15)	0.34^‡^
	Unknown	6	(8.5)	5	(9.8)	1	(5%)	0.67^‡^
Serum TPLA titer, Median, U/mL (IQR)		6571	(2592–15857)	5351	(2325–10774)	14,483	(4054–24596)	0.018^†^
Serum RPR titer, Median, R.U. (IQR)		75.7	(34.4 -188.5)	60.9	(27.8 -136.5)	188.7	(59.8 -337.2)	0.0081^†^
CRP, mg/dL (IQR)		0.23	(0.09–0.65)	0.14	(0.065–0.33)	1.04	(0.61–1.36)	< 0.0001^†^

MLST, multi-locus sequence typing; TPLA, Treponema pallidum latex agglutination; RPR, Rapid Plasma Reagin; CRP, C-reactive protein ^*^ All symptoms were indicative of secondary syphilis and were categorized as such ^†^Mann-Whitney test, ^‡^Fisher’s exact test

Out of the 71cases that were diagnosed with the staging of syphilis, 7 (9.9%) had primary syphilis, 23 (32%) had secondary syphilis, 35 (49%) were diagnosed with early asymptomatic syphilis, and 6 (8.5%) of the cases with second stage syphilis exhibited neurosyphilis. There were no cases of late-stage syphilis.

For cases that were not accessible for MLST, the median RPR was 60.9 (interquartile range (IQR) 27.9-136.5) RU, TPLA was 5351 (IQR 2325–10774) U/mL, and CRP was 0.14 (IQR 0.065–0.33) mg/dL. For fully-typed cases by MLST, the median RPR was 188.7 (IQR 59.8-337.2) RU, TPLA was 14,483 (IQR 4054–24596) U/mL, and CRP was 1.04 (IQR 0.61–1.36) mg/dL. The characteristics of fully-typed cases by MLST were similar to those not accessible for MLST. However, the RPR, TPLA, and CRP levels were significantly higher in the fully-typed cases by MLST group compared to cases not accessible for MLST.

### Multi-locus sequence typing and phylogenetic analysis

**Fig. 1:**
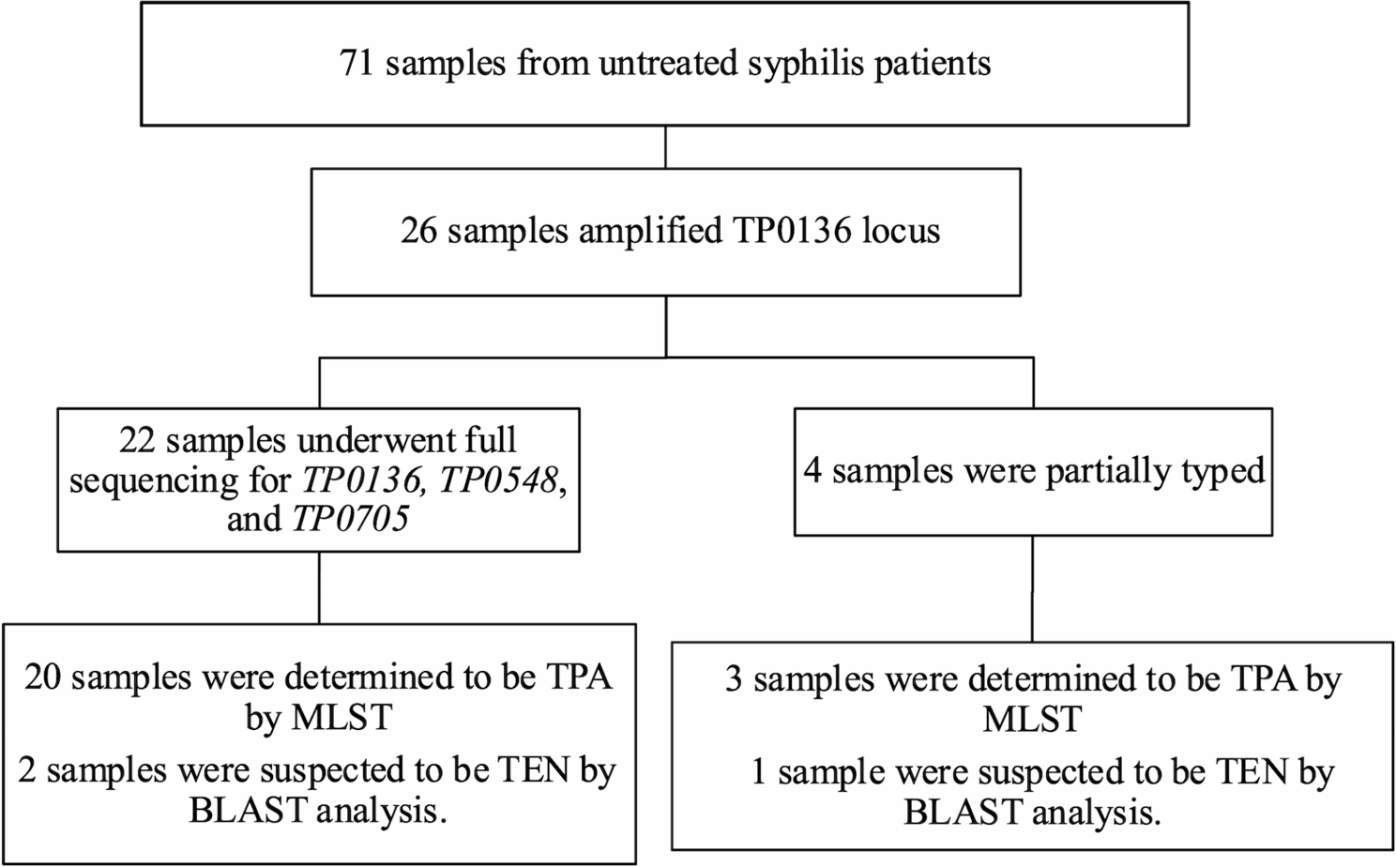
Flowchart of the molecular epidemiology analysis of *Treponema pallidum*. TPA, *Treponema pallidum* subsp. *pallidum*; TEN, *Treponema pallidum* subsp. *endemicum*; MLST, multi-locus sequence typing

**Fig. 2:**
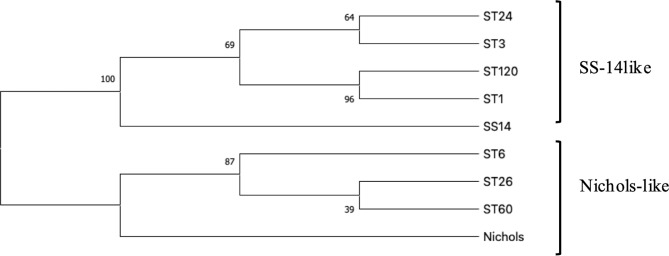
Phylogenetic tree of the identified sequence types from multi-locus sequence typing. The evolutionary history was elucidated using the Maximum Likelihood method. The percentage of trees in which the associated taxa clustered together is presented next to the branches. Initial trees for the heuristic search were automatically generated by applying Neighbor-Join and BioNJ algorithms to a matrix of pairwise distances estimated through the Maximum Composite Likelihood approach

The scheme for detection of *TP0136*,* TP0548*, and *TP0705* and the MLST method is shown in Fig. 1. The profiles of MLST were summarized in Table 2. We found that 26 samples (36.6%) were positive for *TP0136.* However, the full MLST profile was available for 22 samples (28.2%), and a partial profile for 4 samples. In this study, ST3 (1.1.8) (*n* = 9) was predominant, followed by ST6 (3.2.3) (*n* = 6). Additionally, we identified ST60 (9.2.3), ST26 (9.7.3), ST1 (1.3.1), and ST24 (6.1.8). For sample No. 21,100, a new allele was detected on *TP0136* (*TP0136-37*, BIGSdb_20230921112653_3477711_09755) and we applied for a new ST (ST120 [37.3.1]), which was related to ST1(1.3.1) and differed by one substitution. We constructed the phylogenetic tree using the concatenated sequence we detected (Fig. 2). Twelve samples belonged to the SS14-like lineage group (60%), and eight samples belonged to the Nichols-like lineage group (40%). These results indicated that both SS14-like and Nichols-like strains were circulating concurrently in Japanese MSM-PWH. Partial profiles of TPA were obtained for the remaining three cases in Table 2.

**Table 2:** Multi-locus sequence typing profiles of *Treponema pallidum* subsp. *pallidum*

Sample No.	*TP0136*	*TP0548*	*TP0705*	ST	clonal complex
1959	9	2	3	60	Nichols-like
1974	3	2	3	6	Nichols-like
2083	9	7	3	26	Nichols-like
2085	1	3	1	1	SS14-like
2189	3	2	3	6	Nichols-like
2192	1	1	8	3	SS14-like
2193	1	1	8	3	SS14-like
2194	1	1	8	3	SS14-like
2198	3	2	3	6	Nichols-like
21,100	37	3	1	120	SS14-like
21,108	1	1	8	3	SS14-like
21,109	1	1	8	3	SS14-like
21,111	1	1	8	3	SS14-like
21,117	1	1	8	3	SS14-like
21,120	3	*	*		Nichols-like^†^
21,121	3	2	3	6	Nichols-like
21,122	3	2	3	6	Nichols-like
22,126	1	1	8	3	SS14-like
22,129	1	*	*		SS14-like^†^
22,133	3	2	3	6	Nichols-like
22,138	3	2	*		Nichols-like^†^
22,144	6	1	8	24	SS14-like
22,147	1	1	8	3	SS14-like

ST, sequence type, *Sequencing of this allele failed. ^†^ Classification is based on the sequence type for TP0136 only, not the multi-locus sequence typing profile

Two of the 22 samples, in which all three alleles could be sequenced, and one in which only two alleles (*TP0136* and *TP0548*) could be sequenced, could not have the allele numbers determined by MLST. These allelic profiles exhibited unique nucleotide sequences at the typing loci and we referred to BLAST (https://blast.ncbi.nlm.nih.gov/Blast.cgi), and there was a possibility that these three samples (12%, 3/26) were TEN. The reference strain reported from Japan (Japan326e, GenBank: CP073518.1:158259–159364) was used for the identification of TEN [13]. In one case, where all three alleles were successfully sequenced, both *TP0136* and *TP0548* had 100% query cover and 100% identity, with *TP0705* identified as allele number 9. In another case, where all three alleles were sequenced, *TP0136* showed 100% query cover and 100% identity, while TP0548 had 99.91% identity due to a single nucleotide insertion between reference positions 158,578 and 158,579, with *TP0705* again identified as allele number 9. This reference strain also identified TP0705 as allele number 9. In a third case, where only two alleles were sequenced, both *TP0136* and *TP0548* had 100% query cover and 100% identity. None of the three cases had visited tropical regions where bejel is prevalent. Of the three cases with suspected TEN infection, one was diagnosed with secondary syphilis, and the others with early asymptomatic syphilis.

### Investigation of biomarkers and stage of syphilis associated with the *TP0136* detection rate

We examined the factors influencing the detection rate of TPA-derived DNA in whole blood samples (refer to Additional Table for clinical data on *TP0136*-detected samples). In Table 3, no significant association between syphilis stage and *TP0136* detection was observed. However, TP0136 detection rates were 50% (15/30) and 27% (11/41) for symptomatic and asymptomatic syphilis, which includes early asymptomatic syphilis and cases with an unknown stage, respectively (*P* = 0.052).

The RPR, TPLA, and CRP levels were all significantly higher in the *TP0136* detected group compared to the *TP0136* not detected group. Meanwhile, the *TP0136* detection rate and TPLA did not show a statistically significant correlation with cut-offs of 1000 U/mL (*p* = 0.25) or 5000 U/mL (*p* = 0.070). The Cochran-Armitage test for trend also failed to reveal an increase in *TP0136* detection as TPLA levels increased (*p* = 0.056, Table 3). Similarly, the *TP0136* detection rate did not exhibit a significant association with the RPR cut-offs of 10 RU (*p* = 0.40) or 100 RU (*p* = 0.21). The trend test did not show an increase in *TP0136* detected as the RPR increased (*p* = 0.12, Table 3).

**Table 3:** Characteristics of cases that *TP0136* was detected

		All cases		*TP0136* not detected		*TP0136* detected		*P* value
Number of cases		71		45		26		
Stage of syphilis, n (%)	Primary syphilis	7	(9.9)	3	(6.6)	4	(15)	0.41^‡^
	Secondary syphilis	23	(32)	12	(27)	11	(42)	0.19
	Early asymptomatic syphilis	35	(49)	26	(58)	9	(35)	0.085^‡^
	Neurosyphilis^*^	6	(8.5)	2	(4.4)	4	(15)	0.18^‡^
	Unknown	6	(8.5)	4	(8.9)	2	(7.8)	0.99^‡^
Serum TPLA titer,	Median, U/mL (IQR)	6571	(2582–15857)	5209	(2042–9858)	11,262	(4527–23081)	0.0071^†^
	< 1000 U/mL, n	7		6		1		0.056^§^
	1000–5000 U/mL, n	23		16		6		
	> 5000 U/mL, n	41		23		19		
Serum RPR titer	Median, RU (IQR)	75.7	(34.4 -188.5)	66.9	(27.3–136)	115	(52.5–317)	0.016^†^
	< 10 RU, n	6		5		1		0.12^§^
	10–100 RU, n	36		25		12		
	> 100 RU, n	29		15		13		
CRP	Median, mg/mL (IQR)	0.23	(0.09–0.65)	0.13	(0.06–0.31)	0.865	(0.575–1.29)	< 0.0001^†^
	< 0.1 mg/dL, n	19		18		1		< 0.0001^§^
	0.1–0.5 mg/dL, n	26		21		5		
	> 0.5 mg/dL, n	26		6		20		

TPLA, Treponema pallidum latex agglutination; RPR, Rapid Plasma Reagin; CRP, C-reactive protein ^*^ All symptoms were indicative of secondary syphilis and were categorized as such ^†^Mann-Whitney test, ^‡^Fisher’s exact test, ^§^Cochran-Armitage test for trend

In contrast, positive CRP levels were significantly associated with *TP0136* detection at cut-offs of 0.1 mg/dL (*p* = 0.0007) or 0.5 mg/dL (*p* < 0.0001). For CRP ≥ 0.5 mg/dL, 77.0% were positive for *TP0136*. A trend test further revealed that *TP0136* detection significantly increased as CRP levels increased (*p* < 0.0001).

## Discussion

Two distinct phylogenetic lineages of TPA strains, namely Nichols-like and SS14-like, have been identified [14]. Upon reviewing previously analyzed data, it was observed that the SS14-like lineage predominated among the samples obtained in Japan (refer to PubMLST BIGSdb). A previous study, utilizing Sequence-Based Molecular Typing (SBMT) other than the MLST method, reported greater genetic diversity of *T. pallidum* in the MSM population compared to the heterosexual population. Kojima et al. found that in MSM, 63% were SS14-like, 37% were Nichols-like, whereas in heterosexuals, 100% were SS14-like [15]. Similarly, Kanai et al. reported that in MSM, 75% were SS14-like, 25% were Nichols-like, and 25% were macrolide-resistant, while 100% of the heterosexuals were SS14-like [16]. Given that the predominant demographic in our study comprised MSM, our MLST analysis generally aligns with findings from earlier studies that employed different typing methods. Furthermore, WGS analysis revealed a close relationship between SS14-lineage strains in Japan and China [17]. In our study, ST3 (1.1.8) was predominant among 20 blood samples, followed by ST6 (3.2.3). This aligns with the prevalence of ST3 in China [18]. We also identified a new allele belonging to the SS14 lineage, resulting in a new ST that differed from ST1 (1.3.1) by one substitution on *TP0136*. ST1(1.3.1) was frequently detected in Czech Republic, the Netherlands, France, and Cuba [9, 19–21]. Vrbová et al. conducted a large survey in the Czech Republic from 2004 to 2022, finding that SS14-like strains were predominant [22]. The first group of isolates included profiles ST 1 (1.3.1) and ST 25 (1.26.1), while the second group comprised ST 3 (1.1.8), ST 2 (1.1.1), and ST 11 (1.1.3). The two groups accounted for 57.5% and 25.3% of the total isolates, respectively. In our study, ST 3 (1.1.8), which represented 45% of the isolates, belongs to the second group found in the Czech Republic, suggesting a somewhat different predominant strain. The most common Nichols-like strain in the Czech Republic, ST 6 (9.7.3), accounted for only about 6% of the total in the Czech Republic [22], whereas in our study, the major Nichols-like strain, which is also ST 6 (9.7.3), represented 40% of the total, highlighting a significant difference in strain distribution. In summary, our results indicate an MLST profile similar to that of China, considering the geographical proximity to Japan. However, it’s important to note that our typing utilized only three loci, and there might be additional diversity within the SS14-lineage. Limited data for the Asian region in the database highlights the need for a more convenient method to collect data for detailed analysis.

We identified three cases strongly suspected to be caused by TEN, the pathogen associated with bejel. A recent study by Kawabata et al. conducted a sequence-based molecular epidemiological analysis of TPA, which is prevalent in Japan. They encountered instances of TPA that are not easily typed from specimens derived from MSM. Utilizing phylogenetic tree analysis with sequences from *TP0548* [23] and *TP0856* genes [24], which exhibit relatively low homology between TPA/TPE/TEN, they concluded that seven out of 70 cases were infected with TEN [25]. This report covers specimens collected from 2014 to 2019. When combined with the results of our study from 2019 to 2022, it strongly suggests that TEN, traditionally considered a non-sexually transmitted treponema, has been prevalent among Japanese MSM for at least a decade, following a transmission pattern similar to that of TPA in sexually transmitted syphilis.

We were able to detect *TP0136*,* TP0548*, and *TP0705* in approximately one-third of the RPR-positive cases. According to a previous study, the PCR-positive rate when using blood samples was lower than that of swab samples [8]. However, in this study the *TP0136* detection rate, encompassing the partial profile, was 32.9% among TPA. Importantly, this doesn’t imply that whole blood is unsuitable for molecular epidemiological analysis of syphilis. It’s essential to recognize that the gene detection rate serves as a clinical diagnosis within the population of RPR positives. Whole blood samples can easily be collected from all people with syphilis, and the higher sensitivity of PCR positivity in participants with elevated CRP levels suggests an efficient molecular epidemiological analysis by selectively choosing patients for whole blood samples. In this study, we used *TP0136* to screen for PCR positivity as part of an efficient MLST method. However, some studies have utilized multiple alleles or shorter amplicons, such as *polA*, for the genetic diagnosis of syphilis [26, 27]. Vrbova et al. performed PCR on multiple alleles simultaneously and reported positive results in 34.8% of cases [28], which is comparable to the sequencing success rate for three alleles in this study. Wang et al. reported PCR positivity rates of 7.4% in latent syphilis and 62.9% in secondary syphilis [27]. In contrast, our study found no significant difference in detection rates between latent and secondary syphilis; however, differences in HIV status, MSM status, and other background factors between the study populations may have influenced the results.

Moreover, diagnosing and treating infectious diseases based on antibody titers, monitored through before-and-after comparisons, relies on relative evaluations. However, antibody titers take time to decline, posing challenges in determining whether the decline is a treatment effect or a natural process. Given that many syphilis cases are asymptomatic, and individuals who test RPR-positive may have already healed spontaneously, relying solely on antibody titers might not accurately reflect the disease status. Therefore, *T. pallidum* DNA detection rate may offer insights into some etiologies of the disease beyond the sensitivity of the test. A previous study explored the correlation between RPR titer and *T. pallidum* DNA detection rate, considering the syphilis stage [27]. In addition to RPR, we examine the association between PCR-positives and CRP values. CRP, as an indicator of inflammation [29], a typically remains below 0.3 mg/dL in most healthy individuals, with normal or minor elevations falling within the range of 0.3-1.0 mg/dL [30]. This observation may be linked to the bacterial load in the blood during early syphilis, serving as a potential pathogenetic factor alongside technical detection sensitivity. PCR-negatives may not necessarily indicate the absence of the pathogen, and therefore, the need for treatment cannot be ruled out. However, it’s important to note that antibody titers do not necessarily imply the presence of the pathogen. While pathogen testing is fundamental for treating infectious diseases, it has proven insufficient in syphilis. At this stage, it is difficult to use the results of genetic testing of blood samples to make clinical decisions, and further research is needed. However, in the field of syphilis care, there is a need for diagnosis and treatment based on antigen testing rather than antibody titers, as is commonly done for other infectious diseases.

The small sample size is one of the limitations of this study. The uniqueness of this study lies in its use of blood specimens from all stages of the disease, not limited to primary syphilis. In MSM, syphilis often presents only as a rosacea or remains asymptomatic, with only a small percentage exhibiting the skin symptoms characteristic of primary syphilis. However, this study demonstrates the feasibility of conducting molecular epidemiological analysis with blood specimens from any stage of the disease. With a larger sample size, statistical significance could have been established in the relationship between the RPR and *TP0136* detection rate. Nonetheless, numerous cases exhibited low RPR levels with positive PCR results, enabling MLST. The observed discrepancy between the antibody response and the onset of clinical symptoms is a common phenomenon in infectious diseases. Therefore, it remains unclear whether the RPR and *TP0136* detection rate hold clinical significance. Another limitation is the lack of comparison between WGS and MLST. However, given the challenges of applying WGS to TPA, a simpler method is required. This study establishes that, at the very least, MLST possesses the discriminatory power to identify novel epidemic strains and outbreaks of bejel among MSM.

## Conclusion

Our study unveiled the dissemination of SS14-like TPA in Japan, with the concurrent detection of Nichols-like strains. CRP emerged as a potential marker for evaluating PCR performance. Individuals diagnosed with syphilis may include those with bejel, and TEN could have been sexually transmitted among Japanese MSM. The MLST scheme, particularly when utilizing blood samples, proves to be valuable for broader research endeavors.

## Electronic supplementary material

Below is the link to the electronic supplementary material.

Supplementary Material 1

## Data Availability

No datasets were generated or analysed during the current study.
