# Supplementary material for: Epidemic of multiple Treponema pallidum strains in men who have sex with men in Japan: efficient multi-locus sequence typing scheme and indicator biomarkers
Source: AIDS Res Ther. 2024 Oct 16;21:71. doi: 10.1186/s12981-024-00663-y (PMC11484458; doi:10.1186/s12981-024-00663-y)
Supplement: Supplementary file 1 — Supplementary Material 1 [file 12981_2024_663_MOESM1_ESM.docx]

| Additional Table Clinical data of *TP0136* detected samples | | | | |
| --- | --- | --- | --- | --- |
| Sample No. | Year collected | RPR (R.U.) | TPLA (U/mL) | CRP (mg/dL) |
| 1959 | 2019 | 353.5 | 17836 | 0.59 |
| 1974 | 2019 | 44.2 | 3955 | 0.65 |
| 2083 | 2020 | 72.7 | 6571 | 1.04 |
| 2085 | 2020 | 69 | 32037 | 1.3 |
| 2189 | 2021 | 546 | 56864 | 1.24 |
| 2192 | 2021 | 273 | 19140 | 2.88 |
| 2193 | 2021 | 239.7 | 4087 | 2.13 |
| 2194 | 2021 | 42.8 | 3161 | 0.54 |
| 2198 | 2021 | 617.4 | 23866 | 0.62 |
| 21100 | 2021 | 399.9 | 40117 | 1.03 |
| 21108 | 2021 | 9.7 | 600 | 0.57 |
| 21109 | 2021 | 331.7 | 1101 | 1.79 |
| 21111 | 2021 | 61.5 | 5846 | 1.54 |
| 21117 | 2021 | 724.2 | 41126 | 1.13 |
| 21120 | 2021 | 2065.5 | 15130 | 0.19 |
| 21121 | 2021 | 92.4 | 11130 | 0.35 |
| 21122 | 2021 | 270.3 | 24371 | 1.83 |
| 22126 | 2022 | 137.7 | 25270 | 1.22 |
| 22129 | 2022 | 15.6 | 11393 | 0.06 |
| 22133 | 2022 | 249.9 | 3135 | 0.61 |
| 22138 | 2022 | 57.3 | 8858 | 0.12 |
| 22144 | 2022 | 22.5 | 1222 | 0.13 |
| 22147 | 2022 | 29.4 | 20725 | 0.79 |
| RPR, Rapid Plasma Reagin; TPLA, Treponema pallidum latex agglutination; CRP, C-reactive protein | | | | |
